# CXCR4-Overexpressing Umbilical Cord Mesenchymal Stem Cells Enhance Protection against Radiation-Induced Lung Injury

**DOI:** 10.1155/2019/2457082

**Published:** 2019-02-05

**Authors:** Chunyang Zhang, Yan Zhu, Ji Wang, Lisheng Hou, Wei Li, Huaijie An

**Affiliations:** ^1^Respiratory Department of Navy General Hospital, Beijing 100048, China; ^2^Respiratory Department of International Hospital of Zhejiang University, Hangzhou 310009, China; ^3^Health Care Cadre Department of Navy General Hospital, Beijing 100048, China; ^4^Orthopedics Department of Navy General Hospital, Beijing 100048, China; ^5^Center of Basic Medical Sciences of Navy General Hospital, Beijing 100048, China

## Abstract

Less quantity of transplanted mesenchymal stem cells (MSCs) influences the therapeutic effects on radiation-induced lung injury (RILI). Previous studies have demonstrated that MSCs overexpressing Chemokine (C-X-C motif) receptor 4 (CXCR4) could increase the quantity of transplanted cells to local tissues. In the present study, we conducted overexpressing CXCR4 human umbilical cord mesenchymal stem cell (HUMSC) therapy for RILI. C57BL mice received single dose of thoracic irradiation with 13 Gy of X-rays and then were administered saline, control HUMSCs, or CXCR4-overexpressing HUMSCs via tail vein. Transfection with CXCR4 enhanced the quantity of transplanted HUMSCs in the radiation-induced injured lung tissues. CXCR4-overexpressing HUMSCs not only improved histopathological changes but also decreased the radiation-induced expression of SDF-1, TGF-*β*1, *α*-SMA, and collagen I and inhibited the radiation-induced decreased expression of E-cadherin. Transplanted CXCR4-overexpressing HUMSCs also could express pro-SP-C, indicated adopting the feature of ATII. These finding suggests that CXCR4-overexpressing HUMSCs enhance the protection against RILI and may be a promising strategy for RILI treatment.

## 1. Introduction

Radiotherapy, the commonly used treatment for thoracic malignant tumor, can lead to severe complications in some patients, like radiation-induced lung injury (RILI). RILI may include early radiation-induced pneumonitis and advanced stage radiation-induced lung fibrosis [1]. They can affect the patient's quality of life with severe adverse complications [2]. Radiation-induced pneumonitis and lung fibrosis are mostly observed in 4 to 30 weeks and 6 to 12 months after thoracic radiotherapy [3]. The incidence rate of severe irradiation-induced lung injuries reported is 15% among patients treated by thoracic radiotherapy [4]. These patients are presented with symptoms such as cough, panting, dyspnea, and even respiratory failure [5, 6]. As there were few efficient therapeutic methods available for RILI in clinical practice [7], it is critical to understand the complications in great depth and investigate better methods of protection to improve patients' life quality.

MSCs are able to ameliorate various kinds of lung injury and are intensively investigated to develop a therapeutic approach for treating lung diseases such as RILI, idiopathic pulmonary fibrosis (IPF), and emphysema which are lacking a specific and effective therapy [8, 9]. It has been observed that mesenchymal stem cells (MSCs) could home and engraft within the injured tissues and differentiate into vascular cells and alveolar cells to regenerate the damage in the irradiated mouse lung [10]. In *in vivo* and *in vitro* studies, MSCs were found to alleviate irradiation-induced lung injuries not only by the secretion of cytokines, growth factors, and paracrine molecules but also by immunomodulatory effect. Moreover, they could modulate immune response, attenuate inflammation, and regulate the release of proinflammatory and profibrotic molecules involved in fibroblast proliferation and extracellular matrix excess deposition [2, 11–13]. Some previous studies also demonstrated that MSCs act as gene therapy delivery vehicles and attenuate lung injury through enhancing the target gene expression in specific damaged tissue sites in the lungs [14, 15]. MSCs are currently used as a promising therapeutic candidate for alleviation of RILI.

Contrarily, some studies suggested that the quantity of exogenous MSCs transplanted in the injured lung tissues is so less to influence the biological effects of MSCs [2, 16, 17]. Accordingly, some studies were carried out to increase the quantity of MSCs in the injured tissues and improve their therapeutic effect [18–20]. Recent studies have demonstrated that the homing capability is improved, and the therapeutic effect is increased by enhancing the expression of CXCR4 gene in MSCs [19–21]. CXCR4 is a G protein-linked seven transmembrane spanning receptor that has been identified as a receptor of stromal cell-derived factor-1 (SDF-1) for stem cells [22–24]. Previous studies have identified that CXCR4/SDF-1 axis critically influences the migration and homing capabilities of MSCs [25, 26]. Activated CXCR4/SDF-1 axis could recruit MSCs to injured sites in the lungs and increase the number of cells in the local tissues [25, 26]. Liebler et al. [17] found that preincubation of human bone marrow-derived cells with diprotin A, an inhibitor of CD26 peptidase activity that increases the SDF-1/CXCR4 axis, could enhance the number of transplanted cells retained in the bleomycin-induced injured lung injury in mice model. Other studies also showed that CXCR4-overexpressing human MSCs could correlate with higher engraftment in an injured site [27, 28].

In order to specifically enhance the quantity of transplanted MSCs in injured lung tissues, we transplanted CXCR4-overexpressing HUMSCs transduced by lentiviral vector to irradiate mouse models and identified the efficacy of CXCR4-overexpressing HUMSCs on treating RILI in the present study.

## 2. Materials and Methods

### 2.1. Isolation, Culture, and Passage of Human Umbilical Cord Wharton's Jelly-Derived Mesenchymal Stem Cells (HUMSCs)

All experiments in this study were approved by the Navy General Hospital Ethical Review Board. Human umbilical cords were obtained from healthy and full-term births by cesarean section in accordance with the ethical standards of the local ethics committee. Under sterile conditions, the Wharton's jelly was isolated from the umbilical cords and was cut into small pieces of about 1 mm. The cord pieces were then placed in T75 culture flasks with 2.5–3 ml of low-glucose Dulbecco's modified Eagle medium (DMEM; Gibco, USA) supplemented with 10% fetal bovine serum (FBS) (Gibco, USA), 2 mM L-glutamine (Hyclone, USA), 100 IU/ml penicillin (Hyclone, USA), and 100 *μ*g/ml streptomycin (Hyclone, USA) at 37°C in a humidified atmosphere containing 5% CO_2_, and the culture medium was changed after every 3-4 days. The remaining fragments of the Wharton's jelly were wiped off from the flask after 2 weeks of culture. The cultured HUMSCs had attached to the flask surface and reached confluence. When the primary HUMSCs reached about 80% confluence, they were subcultured and cultivated for up to 3–6 passages. The passage 3 cells were used in the present study.

### 2.2. Immunophenotypes of HUMSCs

The passage 3 HUMSCs grown as monolayers were treated with trypsin (Invitrogen, USA) and washed with PBS and staining buffer containing 4% FBS and 0.1% azide in PBS. Every tube contained 1 × 10^6^ HUMSCs for the studies. The HUMSCs were stained with phycoerythrin- (PE-) labeled monoclonal antibodies against human CD29, CD90, and HLA-DR; fluorescein- (FITC-) labeled monoclonal antibodies against human CD34, CD44, and CD45; and APC-labeled monoclonal antibodies against human CD31, respectively (Biosynthesis Biotechnology, Beijing, China). The cells were kept in dark for 30 min at room temperature. The cell's immunophenotypes were detected by flow cytometry (FACS Calibur, Becton-Dickinson, USA). Flow cytometric analyses showed positive reactions for CD29, CD 44, and CD90 but negative for CD31, CD34, CD45, and HLA-DR.

### 2.3. Construction and Transfection of Lentivirus

The overexpression of CXCR4 and EGFP was constructed by lentivirus (GeneChem Co. Ltd., Shanghai, China). The human CXCR4 (gene ID: 7852, NM: 001008540) was cloned into the multicloning site of a lentivirus vector plasmid pGC-FU-3FLAG-SV40-EGFP (GeneChem Co. Ltd., Shanghai, China). The lentivirus-CXCR4-EGFP vector (LV-CXCR4-EGFP) was mixed with pHelper 1.0 and pHelper 2.0, and then was cotransfected into HEK-293T cells with lipofectamine TM2000 (Invitrogen, Shanghai, China) according to the manufacturer's instruction. After 48 h transfection, viral supernatants were collected, filtered through 0.45 *μ*m polyvinylidene fluoride membranes, and then centrifuged. The recombinant lentiviral vector expressing EGFP alone (LV-EGFP) was constructed as control.

We explored the different multiplicities of infection of lentiviral vectors referring some previous studies [15]. In preliminary experiments, the transfected HUMSCs exhibited decreased EGFP expression at low multiplicity of infection (MOI); the morphological characteristics of HUMSCs were changed and the mortality rate was increased at high MOI. We selected the best MOI through evaluating the expression of EGFP, the efficiency of transfection, and the state of HUMSC growth. The passage 3 HUMSCs were transfected with LV-CXCR4-EGFP vectors at MOI of 20 and with LV-EGFP vectors at MOI of 10 and were cultured for following examinations and cell transplantation. The different target genes carried by either LV-CXCR4-EGFP vector or LV-EGFP vector perhaps made the difference of two MOIs.

After 96 hours, HUMSCs transfected with LV-CXCR4-EGFP or LV-EGFP were examined through a fluorescence microscope. The overexpression of the CXCR4 gene was detected by western blotting and immunofluorescence.

### 2.4. MTT Assay

To observe the effect of CXCR4 overexpression on HUMSCs, normal, control, and CXCR4-overexpressing HUMSCs were, respectively, seeded in 96-well plates (2 × 10^4^ cells per well) and incubated at 37°C for 4 h in an incubator containing 5% CO_2_, with 20 *μ*l of 3-(4,5-dimethylthia-zol-2-yl)-2,5-diphenyltetrazolium bromide (MTT). Subsequently, the CM and MTT were removed, and 150 *μ*l of dimethyl sulfoxide (DMSO) added to each well. The samples were then shaken, and the optical density (OD) values at 570 nm measured using an ELISA meter (3001-122J, Thermo, USA). Assays were performed on days 0, 2, 4, 6, and 8 after transfection. Experiments were performed 6 times.

### 2.5. Migration Assay

HUMSCs (1 × 10^4^/well) were cultured in the polycarbonate membrane of the upper transwell chambers (8 *μ*m pores/24 wells; collagen-coated; Corning Costar, Corning, USA) in DMEM/F12 for 12 hours at 37°C in an incubator containing 5% CO_2_, where the under transwell chambers contained SDF-1 (100 ng/ml, SAB, USA, 801121) in DMEM/F12 or kept blank. Then the residual HUMSCs on the upper side of the polycarbonate membrane were removed. The membranes were rinsed in paraformaldehyde for 15 min and then the cells were stained with hematoxylin. To quantify the migration of transfected HUMSCs, the number of cell on the underside of the membrane was determined from 5 randomly selected areas per field (magnification, ×200) with microscope.

### 2.6. Animal Model Construction and HUMSC Transplantation

Seventy-two female C57BL mice of 2 months old (from Military Medical Science School, Beijing) were procured and acclimated for one week before conducting the study. All experiments and procedures were approved by the Committee on the Ethics of Animal Care and Use of Navy General Hospital. The mice were randomized into 4 groups: normal, radiation, control, and CXCR4-overexpressing. Before irradiation, 5 mice were subjected to CT scan to determine the size of the lungs, and linear accelerator (oncor impression, Siemens) was adjusted to restrict X-rays to just the thorax. The mice, except the normal group, received single dose of thoracic irradiation (13 Gy, 6 MV, 3.68 Gy/min, window width 20 × 2.5 cm) using the linear accelerator. Saline (200 *μ*l), control HUMSCs (5 × 10^5^/200 *μ*l), or CXCR4-overexpressing HUMSCs (5 × 10^5^/200 *μ*l) was injected intravenously via the tail vein, one day after radiation, respectively. Mice were killed on day 7, 30, or 60 after treatment. The right lungs were used for RNA and protein extraction; the left lungs were fixed in 10% paraformaldehyde for histopathological analysis.

### 2.7. Lung Morphology and Immunohistochemistry

The samples from left lungs were embedded by paraffin and were sectioned at a thickness of 5 *μ*m. And then they were stained with hematoxylin and eosin (HE) and Masson, respectively. Lung pathology was detected by light microscopy. After deparaffinization, the paraffin-embedded lung sections were incubated with rabbit polyclonal anti-collagen antibody (1 : 200, Abcam, USA, ab34710), rabbit polyclonal anti-*α*-SMA antibody (1 : 250, Abcam, USA, ab5694), or rabbit polyclonal anti-E-cadherin antibody (1 : 50, Abcam, USA, ab15148) antibody, respectively, for 2 h at 37°C, followed by an incubation with goat–anti-rabbit IgG-HRP antibody (1 : 1000, Sigma-Aldrich, USA, A6154) for 40 min at 37°C. 3,3-diaminobenzidine (DAB) was used as chromogenic substrate.

### 2.8. Immunofluorescence

The CXCR4-overexpressing HUMSCs or control HUMSCs, grown on glass coverslips, and frozen mouse lung tissues, sectioned at 4 *μ*m and placed onto glass slides, were incubated overnight with rabbit polyclonal anti-CXCR4 antibody (1 : 200, Boster, China, BA0761), or rabbit polyclonal anti-pro-SP-C antibody (1 : 250, Abcam, USA, ab40879), respectively, in darkness at 4°C, followed by incubations with Dylight 549 (1 : 100, Abbkine, USA, A23320) for 1 h at 37°C. Then the total cell nuclei of the HUMSCs and the lung tissues were stained with 4′,6-diamidino-2-phenylindole (DAPI) (Sigma-Aldrich). The fluorescence images of EGFP, CXCR4, and pro-SP-C were detected using a confocal laser scanning microscope (Leica, Germany, TCS SP8).

### 2.9. Western Blot

HUMSCs, lysed in RIPA buffer, and frozen lung tissues, ground in a mortar with liquid nitrogen, were all stored aliquots at −80°C until assayed, respectively. Lysates were mixed with an equal volume of sample buffer, denatured by boiling, and then separated on a 10–15% polyacrylamide minigel. The proteins were transferred to nitrocellulose membranes, blocked with 5% milk, and incubated overnight with rabbit polyclonal anti-CXCR4 antibody (for HUMSC sample, 1 : 100, Boster, China, BA0761), mouse monoclonal anti-CXCR4 antibody (for lung tissue samples, 1 : 500, Abcam, USA, ab58176), or mouse monoclonal anti-TGF-*β*1 antibody (1 : 1000, Abcam, USA, ab64715) at 4°C. And then the blots were incubated with goat-anti-rabbit IgG HRP antibody (1 : 1000, Applygen Technologies Inc., China, C1309) or goat-anti-mouse IgG-HRP antibody (1 : 5000, Abcam, USA, A4416) for 1 h at room temperature. The membranes were rinsed with washing buffer and incubated with chemiluminescence (ECL) working solution for 5 min. The signals were detected by Gel software, and the relative intensities of the detected bands compared between groups.

### 2.10. RT-PCR Examination to Detect Engrafted HUMSCs in Mouse Lung

Lung samples underwent mRNA extraction using RNAprep pure tissue kit (TIANGEN Biotech, Beijing, China, DP431). For *β*-actin gene, the forward primer sequence was 5′-CTGGAACGGTGAAGGTGACA-3′ and the reverse primer sequence was 5′-AAGGGACTTCCTGTAACAATGCA-3′ (GenBank accession number: NM_001101), present in humans and not in mouse to detect the human cellular cDNA in samples. Reverse transcription polymerase chain reaction (RT-PCR) analysis for the human marker, *β*-actin gene, was performed using primers that span the gene (94°C 5 min, 94°C 30 seconds, 58°C 30 seconds, 72°C 30 seconds, 72°C 7 min, 4°C ∞). PCR products were detected by the ethidium bromide-stained 2.5% agarose gel.

### 2.11. Cytokine Examination by ELISA

The SDF-1 protein in the lung homogenate was quantified by ELISA according to the manufacturer's instructions (Abcam, USA).

### 2.12. Statistical Analysis

Values are presented as the mean ± standard deviation (SD). Multiple groups were compared with a Tukey-Kramer post hoc test after an analysis of variance (ANOVA). Two continuous variables were compared using Student's *t*-test. Differences between groups were considered to be significant at *P* < 0.05.

## 3. Results

### 3.1. Characterization of HUMSCs

Adherent HUMSCs were present around the Wharton's jelly fragments after 10 days of culture. Most of the HUMSCs appeared spindle-shaped under light microscopy, and after 3 weeks of culture, the quantity of HUMSCs increased and they aggregated like a vortex (Supplementary Figure 1(a)). A flow cytometric analysis presented that HUMSCs were positive for CD29, CD44, and CD90 and were negative for CD31, CD34, CD45, and HLA-DR, which is consistent with previous reports [29–31] (Supplementary Figure 1(b)). As described previously, HUMSCs in this study had the capacity to differentiate into osteoblasts, chondrocytes, and adipocytes [29]. The results indicated that the cultured cells had the characteristic of mesenchymal stem cells and differ from hematopoietic cell lineage and endothelial progenitor cell lineage.

### 3.2. Effect of CXCR4 Overexpression on HUMSCs' Proliferation, Migration, and Distribution

The MTT assay was used to observe the effects of CXCR4 overexpression on the proliferation of HUMSCs. The HUMSCs were transfected with LV-CXCR4-EGFP vectors or LV-EGFP vectors, and observations made 0, 2, 4, and 6 d after transfection. Measurement of OD values showed that the proliferation of HUMSCs in control and CXCR4-overexpressing group was all increased from day 0 to day 6. There was no significant differences between the CXCR4-overexpressing group or control group and normal group in the OD values, respectively (*P* > 0.05, *n* = 6). This reflects the normal proliferative capacity of these cells in the CXCR4-overexpressing group and control group (Supplementary Figure 3).

**Figure 1:**
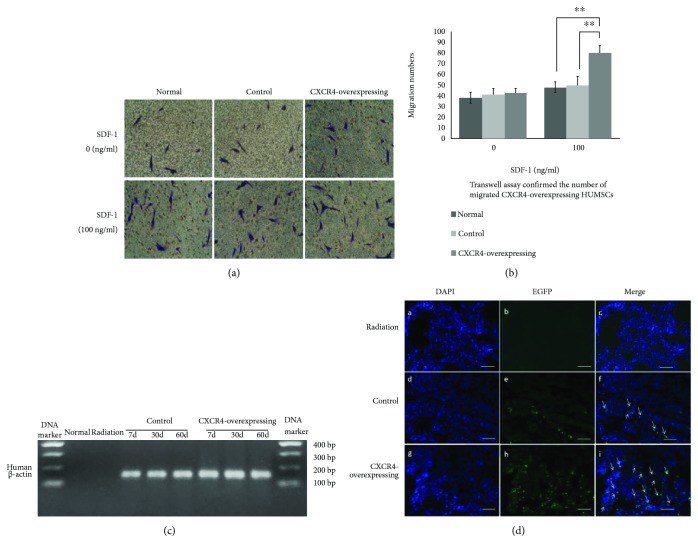
Effect of CXCR4 overexpression on HUMSCs' migration and distribution. (a) The migrated HUMSCs in the down side of the polycarbonate membrane in transwell by hematoxylin staining under white light microscopy at 200x magnification. (b) Transwell assay confirmed that the number of migrated CXCR4-overexpressing HUMSCs was increased compared to control and normal HUMSCs when the under chambers contained SDF-1 (^∗∗^ *P* < 0.01, *n* = 5). There was no difference between every group not containing SDF-1. (c) Examination of human *β*-actin expression in mouse lung tissues by detecting cDNA with hACTB-specific primers. The expression of *β*-actin only present in human but not in mouse was detected in control and CXCR4-overexpressing groups and was absent in normal and radiation groups. (d) The EGFP-positive cells were detected by the fluorescence examination in frozen lung sections. The quantity of EGFP-positive cells was much more in the CXCR4-overexpressing group compared to control group (DAPI: blue; EGFP: green).

To determine whether the migration capacity of CXCR4-overexpressing HUMSCs increased after transfection, we quantified the number of migrated normal, control, and CXCR4-overexpressing HUMSCs in transwell assay *in vitro*. It showed that the number of migrated CXCR4-overexpressing HUMSCs was significantly increased compared to control and normal HUMSCs when the under transwell chambers contained SDF-1 (*P* < 0.001, CXCR4-overexpressing HUMSCs: 80 ± 7 vs. control HUMSCs: 50 ± 8, normal HUMSCs: 48 ± 5). There was no significant differences between the three groups in the migration numbers when the under transwell chambers were without SDF-1 (*P* > 0.05, CXCR4-overexpressing HUMSCs: 43 ± 4 vs. control HUMSCs: 41 ± 6, normal HUMSCs: 38 ± 5) (Figures 1(a) and 1(b)). These results confirmed that the transduced HUMSCs expressing CXCR4 enhanced the migration capacity, which is consistent with previous reports [32, 33].

In order to investigate whether HUMSCs could home to the injured lung and transduction of CXCR4 could enhance homing and migration capacities of HUMSCs *in vivo*, we performed RT-PCR examination and immunofluorescence assays. As previous study described, we amplified sequences of *β*-actin gene (GenBank accession number: NM_001101) only present in human but not in mouse by reverse transcriptase PCR to determine the successful transplantation of HUMSCs into mouse lung [20, 34]. The expression of *β*-actin in mouse lung tissues was observed in the CXCR4-overexpressing group and control group but not in normal group and radiation groups (Figure 1(c)), which indicated that CXCR4-overexpressing HUMSCs and control HUMSCs had been successfully transplanted into the mouse and migrated to the irradiated lung tissue.

The fluorescence examination showed that EGFP-positive cells were observed in frozen lung sections of the mice in the CXCR4-overexpressing and control groups on day 7 postirradiation. Moreover, the quantity of EGFP-positive cells was significantly higher in the CXCR4-overexpressing group compared to control group (Figure 1(d)).

The above results indicated that the transplanted HUMSCs smoothly immigrated to the injured lung tissues after irradiation, and the HUMSCs transduced by lentiviral vector overexpressing CXCR4 gene distributed more in lung tissues than in control HUMSCs.

### 3.3. CXCR4 Gene Overexpression in HUMSCs and Lung Tissue and Effect on SDF-1 Expression

To identify lentivirus transfection, which can overexpress CXCR4 gene, the expressions of EGFP and CXCR4 were examined by fluorescence microscopy, immunofluorescence, and western blots. After 96 h transfected with LV-CXCR4-EGFP or LV-EGFP, the green fluorescence of EGFP was detected in CXCR4-overexpressing and control HUMSCs by fluorescence microscope. The transfection efficiency was above 80% (Supplementary Figure 2), and the green fluorescence of EGFP was still detected after normal subculturing of the transduced HUMSCs.

**Figure 2:**
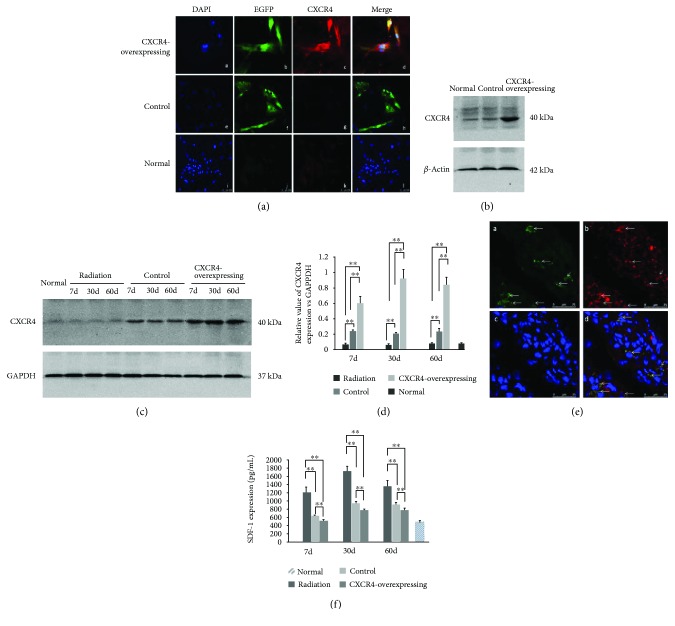
CXCR4 overexpression in HUMSCs and lung tissue and effect on SDF-1 expression in lung tissue. (a) Expressions of EGFP and CXCR4 were examined by immunofluorescence *in vitro* (DAPI: blue; EGFP: green; CXCR4: red). (b) CXCR4 protein was assessed by western blot analysis in HUMSCs. Transfection increased the expression of CXCR4 in CXCR4-overexpressing HUMSCs. (c) CXCR4 protein was assessed by western blot analysis in mouse lung tissue. (d) Densitometric quantification of CXCR4 protein levels in lung tissues of the control and CXCR4-overexpressing groups. Expression of CXCR4 markedly increased in the CXCR4-overexpressing group (^∗∗^ *P* < 0.01, *n* = 6). (e) Immunofluorescence identified EGFP and CXCR4 expressions in transplanted CXCR-overexpressing HUMSCs in mouse lung tissue (DAPI: blue; EGFP: green; CXCR4: red). (f) Expression of SDF-1 in the lung tissue was measured by ELISA. Compared to control group, the radiation-induced increased expression of SDF-1 was obviously inhibited in the CXCR4-overexpressing group (^∗∗^ *P* < 0.01, *n* = 6).

The immunofluorescence and western blotting demonstrated that the CXCR4 protein expression was higher in CXCR4-overexpressing HUMSCs compared to control and normal HUMSCs *in vitro* (Figures 2(a) and 2(b)). These results confirmed that HUMSCs had transducted with LV-CXCR4-EGFP or LV-EGFP and significantly expressed the target gene.

Western blot was used to detect the expression of CXCR4 in lung tissue on days 7, 30, and 60 after radiation. We could not find obvious CXCR4 expression in the normal and radiation groups, whereas in the CXCR4-overexpressing and control groups, the expression of CXCR4 was observed. A negligible expression of CXCR4 in the control group was observed, which could be due to the transplanted HUMSCs naturally expressing CXCR4 (Figure 2(c)). Western blot analysis showed that CXCR4-overexpressing group exhibited much higher CXCR4 expression, as compared to control group (Figure 2(d)). The immunofluorescence also identified the expression of CXCR4 in the EGFP-positive cells of CXCR4 group lung tissue on day 7 postirradiation (Figure 2(e)).

To determine the effects of HUMSCs on SDF-1 levels, ELISA was performed to detect the SDF-1 expression in the lung tissue. The expression of SDF-1 increased on 7 d, 30 d, and 60 d after irradiation, whereas the level of SDF-1 decreased after administration of HUMSCs. CXCR4-overexpressing HUMSCs attenuated the increase of SDF-1 level significantly in contrast to control HUMSCs (Figure 2(f)). These results suggested that CXCR4-overexpressing HUMSCs could significantly inhibit the enhancement of SDF-1 induced by radiation in lung tissues, in contrast to control HUMSCs.

### 3.4. CXCR4-Overexpressing HUMSCs Attenuated Radiation-Induced Lung Injury

**Figure 3:**
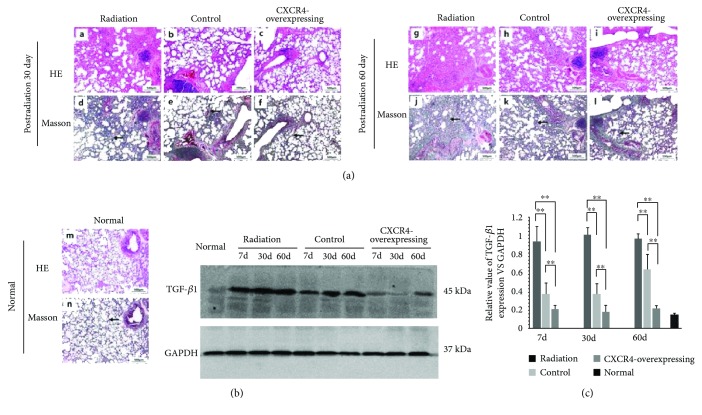
Protective effect of HUMSCs on RILI. (a) Compared to control HUMSCs, CXCR4-overexpressing HUMSCs could attenuate histopathological changes of RILI by hematoxylin and eosin (H&E) and Masson staining of lung sections. (b) TGF-*β*1 protein was assessed by western blot analysis in the mouse lung tissue. (c) Densitometric quantification of TGF-*β*1 protein levels in the lung tissue. The radiation-induced enhancement of TGF-*β*1 was inhibited markedly in the CXCR4-overexpressing group compared to control group (^∗∗^ *P* < 0.01, *n* = 6).

The HE staining of lung sections showed that radiation induced obvious inflammatory infiltrates and alveolar septa thickness on day 30 postirradiation. Aggravated interstitial hyperplasia, interstitial pachy congestion, interalveolar septal thickening, and partial consolidation were observed on day 60. The Masson staining of lung sections showed marked collagen deposition on 30 d and 60 d postirradiation, indicating that transplantation of HUMSCs could attenuate lung injuries. Administration of CXCR4-overexpressing HUMSCs markedly reduced the morphological lung damage compared with control HUMSCs (Figure 3(a)).

TGF-*β*1 is a critical factor in radiation-induced fibrosis [35]. Western blot analysis showed that radiation resulted in a remarkable increase in the level of TGF-*β*1 in the lung tissue of radiation group on 7 d, 30 d, and 60 d postirradiation. Administration of HUMSCs attenuated the radiation-induced elevation of TGF-*β*1 levels. Moreover, the expression level of TGF-*β*1 markedly decreased in the CXCR4-overexpressing group in contrast to control group (Figures 3(b) and 3(c)).

### 3.5. Effect of CXCR4-HUMSCs on *α*-SMA, Collagen I, and E-Cadherin and HUMSCs Adopted ATII Cell Phenotype

**Figure 4:**
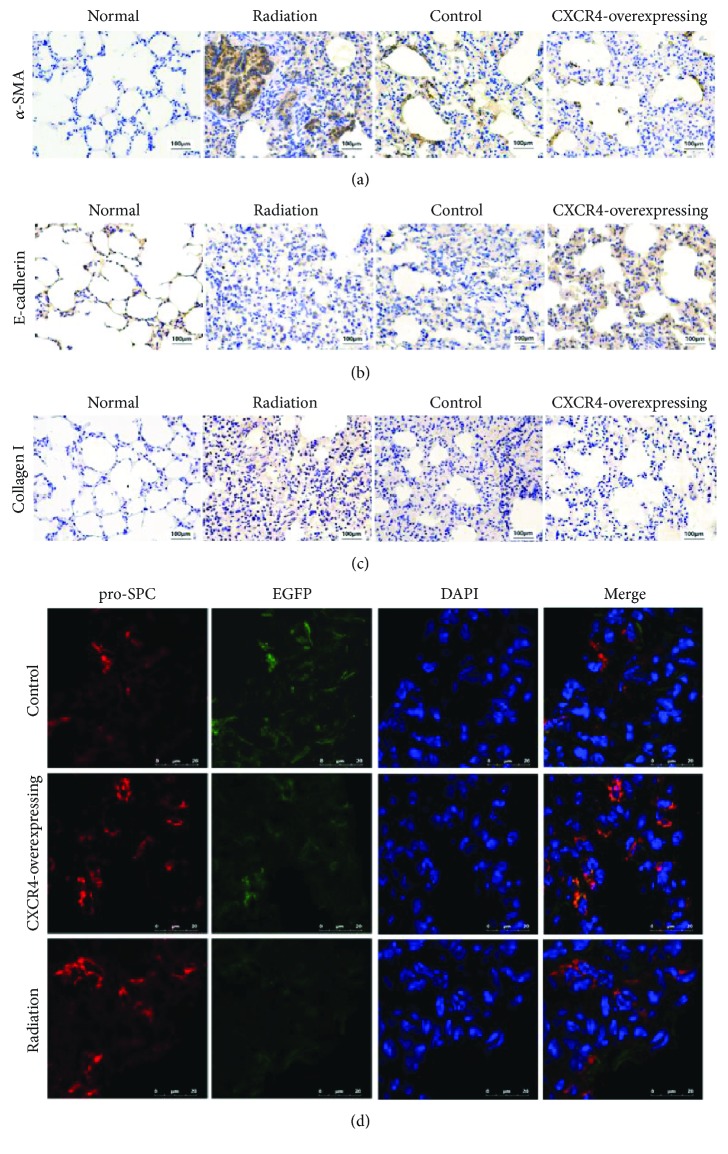
Effect of HUMSCs on *α*-SMA, E-cadherin, and collagen I and HUMSCs adopted alveolar type II cell phenotype. (a) Immunohistochemistry images for *α*-SMA at 30 days postradiation. (b) Immunohistochemistry images for E-cadherin at 30 days postradiation. (c) Immunohistochemistry images for collagen I at 30 days postradiation. (d) Immunofluorescence (IF) of frozen lung sections from control, CXCR4-overexpressing, and radiation groups at 7 days postradiation by using deconvolution microscopy (DAPI: blue; EGFP: green; pro-SPC: red).

As a unique marker of myofibroblasts, *α*-SMA plays a critical role in lung fibrosis and “epithelial-mesenchymal transition” (EMT). A previous study had reported that radiation could induce increased *α*-SMA expression in human lung fibroblasts [5]. In our study, immunohistochemistry analysis demonstrated that *α*-SMA expression was increased in mice lung of the radiation group at 30 days after radiation (Figure 4(a)), whereas the expression of *α*-SMA markedly reduced after the administration of HUMSCs (Figure 4(a)). The *α*-SMA expression was slightly decreased in the CXCR4-overexpressing group compared to control group (Figure 4(a)).

In addition, E-cadherin expression in immunohistochemistry assays showed an obvious decrease at 30 days after irradiation, which exhibited an important role in maintaining the tight junctions of epithelial cells and normal functions of the paracellular barrier in the airway epithelia [5] (Figure 4(b)). However, after administration of HUMSCs, the expression of E-cadherin did not decrease critically in contrast to the radiation group (Figure 4(b)), and the expression of E-cadherin in the CXCR4-overexpressing group was higher than that in the control group (Figure 4(b)).

After irradiation, a significant increase in collagen deposition was reported in pulmonary fibrosis [36]. In this study, the expression of type I collagen was increased at 30 days after irradiation (Figure 4(c)), which was inhibited by administrated HUMSCs (Figure 4(c)). There was no obvious difference in type I collagen expression between the CXCR4-overexpressing HUMSC group and control group.

To confirm whether the transplanted HUMSCs differentiated to alveolar type II (ATII) cells in the mice lung tissue after irradiation, immunofluorescence was performed to detect the expression of pro-SP-C, which is a typical marker of ATII cell differentiation in the mammalian lung [37]. In the control and CXCR4-overexpressing group, the EGFP-positive cells exhibited the expression of pro-SP-C on day 7 after irradiation. This revealed that transplanted HUMSCs could adopt the features of ATII cells in the lung tissue after transplantation (Figure 4(d)).

## 4. Discussion

Recent studies have demonstrated that transplanted MSCs could alleviate the lung injury [2, 11, 15], but the quantity of delivered MSCs in local injured lung tissue was not enough as we anticipated [9, 17, 38–40]. So, we carried out the studies to probe the resolutions for enhancement of homing and retention of stem cells in the injured organs. In this study, CXCR4-overexpressing HUMSCs showed not only an increased capacity of migration *in vitro* but also the enhancement of homing efficiency *in vivo*. Moreover, it showed that CXCR4-overexpressing HUMSCs could alleviate RILI in our mice model.

Previous study showed that 0.1% lung cells were derived from transplanted MSCs [2], and a relatively low frequency of engraftment cells influenced their therapeutic effect [16]. Chang et al. have suggested that homing of culture-expanded MSCs was not as efficient as hematopoietic stem cells and leukocytes for lack of relevant chemokine receptors and cell adhesion [41].

In our study, CXCR4-overexpressing HUMSCs have been shown to be more efficiently retained in postirradiated lung tissues, suggesting that the enhancement of CXCR4 expression in HUMSCs could further improve their homing capacity. Moreover, histopathology and TGF-*β*1 examination showed that CXCR4 overexpression in HUMSCs could alleviate the RILI better in contrast to control HUMSCs.

TGF-*β*1 is one of the important growth factors in the process of RILI [7] and is considered as a master switch for fibrotic program [35]. In clinical practice, the increased TGF-*β*1 level in plasma is the marker of subsequent RILI in patients administrated with radiation therapy [42]. In a previous study, it was shown that the MSCs could decrease the level of TGF-*β*1 [2]. We found that transplanted HUMSCs could inhibit irradiation-induced enhancement of TGF-*β*1, which was consistent with the previous study. Moreover, CXCR4-overexpressing HUMSCs showed better downregulation effect on TGF-*β*1 in contrast to control HUMSCs.

Previous studies have reported that the expression of SDF-1 increased within the injured lung [17, 43]. In the present study, we found that radiation could induce the enhancement of SDF-1 in lung tissues, which was consistent with the previous study [2]. After administration of HUMSCs, we found that HUMSCs could inhibit the enhanced SDF-1 expression in the irradiated lung tissues. Consequently, CXCR4-overexpressing HUMSC delivery had a better effect on the increase of SDF-1 level in this study.

Some studies confirmed that fibrocytes derived from the bone marrow are recruited in the damaged lung tissues and participated in the development of lung fibrosis [44–46]. These circulating fibrocytes could express CXCR4 [47] and are considered to be the resource of lung fibroblasts in response to lung injury in the body through the CXCR4/SDF-1 axis. Moreover, the elevated SDF-1 associates with a large quantity of circulating fibrocytes during the process of pulmonary fibrosis [48]. Song et al. treated bleomycin-induced pulmonary fibrosis mice with AMD3100, a CXCR4 antagonist, to decrease the expression of SDF-1 and circulating fibrocytes in the lung tissue [49]. They used CXCR4 antagonist to inhibit the expression of CXCR4 in circulating fibrocytes and to decrease chemotaxis and recruitment of the circulating fibrocytes to the damaged lung tissues, which resulted in alleviating the lung injury [49]. In our study, we used exogenous CXCR4-overexpressing HUMSCs to attenuate RILI through the enhancement of MSC homing ability. There was no contradiction between our study and the previous study. CXCR4 antagonist treatment could decrease the SDF-1 mRNA expression in the previous study [49], whereas in the present study the SDF-1 level decreased significantly after the administration of HUMSCs. This result indicated that HUMSCs perhaps could decrease the circulating fibrocytes, which contributed to the lung injury and fibrosis, through inhibition of radiation-induced increase of SDF-1. However, CXCR4-overexpressing HUMSCs had a better effect on it in contrast to control HUMSCs in the present study. Additional work is required to elucidate the underlying mechanisms.

The pathologic process of RILI develops through a complex mechanism and involves several cell types in the lung. Fibroblasts and alveolar epithelial cells in the lung are considered to play a key role in the process of RILI [5, 36]. Not only in the differentiation of fibroblasts into myofibroblasts but also in the transition of alveolar epithelial cells to myofibroblasts, the expression of *α*-SMA is increased in both *in vitro* and *in vivo* studies, which is a hallmark of these two transitions [5, 13]. In the process of RILI, myofibroblasts showed increased *α*-SMA expression and synthesized more collagens, which lead to the remodeling of the extracellular matrix. In a previous study, we have identified that irradiation could induce the expression of *α*-SMA enhancement in human lung fibroblasts and result in the differentiation of human lung fibroblasts into myofibroblasts, which is inhibited by coculture with HUMSCs [13]. In the present study, we demonstrated that CXCR4-overexpressing HUMSCs could inhibit the increased expression of *α*-SMA and collagen I in irradiated lung tissues. These results suggested that the transfected HUMSCs could relieve the remodeling of the extracellular matrix.

Alveolar epithelial cell is one of the cell types involved in the process of RILI. The alveolar epithelium injury has been considered to be a key factor in the development of RILI [50]. Alveolar epithelial injury could induce an abnormal epithelial repair, which is a critical pathological feature of lung fibrosis [5]. In the process of RILI, resident epithelial cells undergo transdifferentiation into myofibroblasts, a process termed EMT, which is characterized by increased expression of *α*-SMA and decreased expression of the epithelial marker, E-cadherin [5]. In the present study, irradiation could increase the expression of *α*-SMA and decrease the expression of E-cadherin in lung tissues postirradiation. The decreased expression of E-cadherin also indicated that the tight junctions between alveolar epithelial cells are damaged by irradiation. The abnormal expression of *α*-SMA and E-cadherin in lung tissues postirradiation was alleviated by administrating HUMSCs, which was obviously by transplanted CXCR4-overexpressing HUMSCs. These results showed that CXCR4-overexpressing HUMSCs could perhaps inhibit the process of fibroblast differentiation into myofibroblasts and EMT in lung tissues postirradiation.

Type II alveolar epithelial (ATII) cells have been identified to be the stem cells for producing AEI cells, which are sensitive to injury and incapable of self-renewal. Therefore, treatments that aim to repair or limit epithelial damage might become a key therapeutic strategy to accelerate recovery in lung injury [2, 51]. MSCs have been shown to replace and repair the injured alveolar epithelial cells by differentiation to ATII cells in *in vitro* studies [2, 39, 52–54]. In this study, HUMSCs were found to express pro-SP-C, which was the characteristic of ATII. The mechanism of engrafted MSCs adopting the phenotype of ATII cells remains unclear, which may be related with the differentiation of MSCs to ATII, the cellular fusion or combination between MSCs and resident ATII cells [40, 55]. But some studies showed that transplanted MSCs could not differentiate into lung epithelium and mainly differentiate into myofibroblasts, especially in MSCs administrated late after irradiation [2, 11, 38, 56]. The different microenvironment in early and late lung injury phases may perhaps influence the engrafted MSCs' differentiation [2, 11]. In this study, we have early administrated HUMSCs 1 day after irradiation by the tail vein injection. The involvement of transplanted MSCs' differentiation discrepancy needs further study in the future.

## 5. Conclusions

In conclusion, the present study reveals that HUMSCs, either plain or transfected with CXCR4 gene, could have the therapeutic effects on RILI. CXCR4-overexpressing HUMSCs do have advantages on homing and reparative effects. Thus, therapeutic application of gene-modified HUMSC overexpression of CXCR4 may be helpful for the attenuation of RILI.

## Data Availability

The data used to support the findings of this study are included within the article.

## Supplementary Materials

Supplementary Materials Supplementary Figure 1: characterization of HUMSCs. (a) Morphology of HUMSCs. HUMSCs were around the Wharton's jelly fragments at 21 days of culture at 40x (a) magnification (bar = 100 *μ*m). The passage 3 HUMSCs appeared spindle-shaped and reached confluence circinately after 3 days of culture at 40x (b) (bar = 200 *μ*m), and 100x (c) magnification (bar = 100 *μ*m). (b) Immunophenotype of HUMSCs by flow cytometric analysis. The HUMSCs were positive for CD29, CD44, and CD90 and were negative for CD31, CD34, CD45, and HLA-DR. Supplementary Figure 2: transduction of CXCR4 into HUMSCs using a lentiviral vector. The passage 3 cultured HUMSCs after transfection with lentivirus-CXCR4-EGFP vectors (a) or lentivirus-EGFP vectors (c) under white light microscopy at 200x magnification (bar = 50 *μ*m). The expression of EGFP (green) in HUMSCs detected at 96 h after transduction under fluorescence microscopy at 200x magnification; ((b) lentivirus-CXCR4-EGFP vectors; (d) lentivirus- EGFP vectors; bar = 50 *μ*m). Supplementary Figure 3: MTT assay for HUMSCs of CXCR4-overexpressing, control, and normal (*P* > 0.05, *n* = 6).
