# Supplementary figures and images for: CXCR4-Overexpressing Umbilical Cord Mesenchymal Stem Cells Enhance Protection against Radiation-Induced Lung Injury
Source: Stem Cells Int. 2019 Feb 5;2019:2457082. doi: 10.1155/2019/2457082 (PMC6379846; doi:10.1155/2019/2457082)

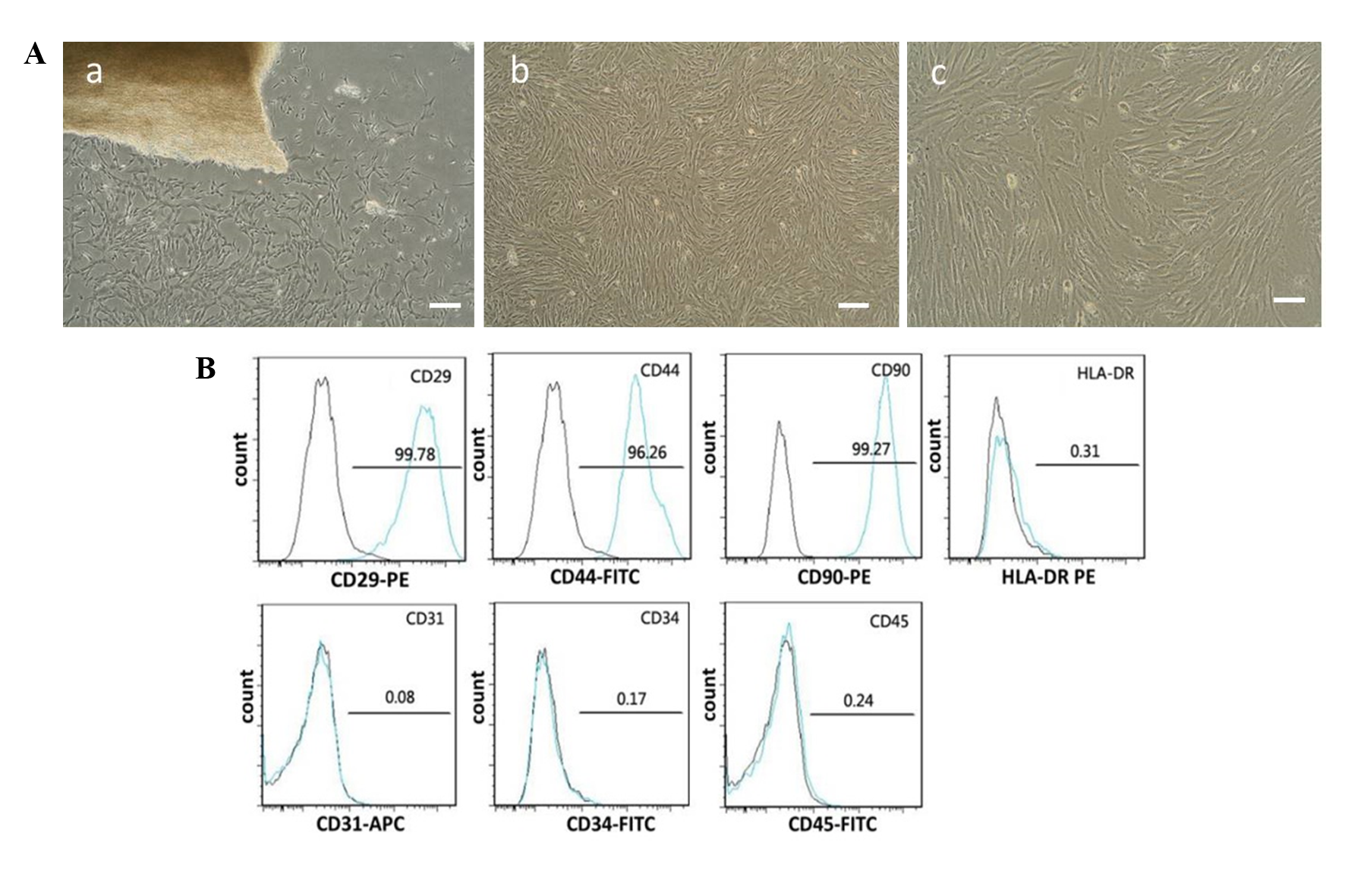

Supplement: Supplementary Materials — Supplementary Figure 1: characterization of HUMSCs. (a) Morphology of HUMSCs. HUMSCs were around the Wharton's jelly fragments at 21 days of culture at 40x (a) magnification (bar = 100 μm). The passage 3 HUMSCs appeared spindle-shaped and reached confluence circinately after 3 days of culture at 40x (b) (bar = 200 μm), and 100x (c) magnification (bar = 100 μm). (b) Immunophenotype of HUMSCs by flow cytometric analysis. The HUMSCs were positive for CD29, CD44, and CD90 and were negative for CD31, CD34, CD45, and HLA-DR. Supplementary Figure 2: transduction of CXCR4 into HUMSCs using a lentiviral vector. The passage 3 cultured HUMSCs after transfection with lentivirus-CXCR4-EGFP vectors (a) or lentivirus-EGFP vectors (c) under white light microscopy at 200x magnification (bar = 50 μm). The expression of EGFP (green) in HUMSCs detected at 96 h after transduction under fluorescence microscopy at 200x magnification; ((b) lentivirus-CXCR4-EGFP vectors; (d) lentivirus- EGFP vectors; bar = 50 μm). Supplementary Figure 3: MTT assay for HUMSCs of CXCR4-overexpressing, control, and normal (P > 0.05, n = 6). [file 2457082.f1.zip › Figure/S Fig1.tif]

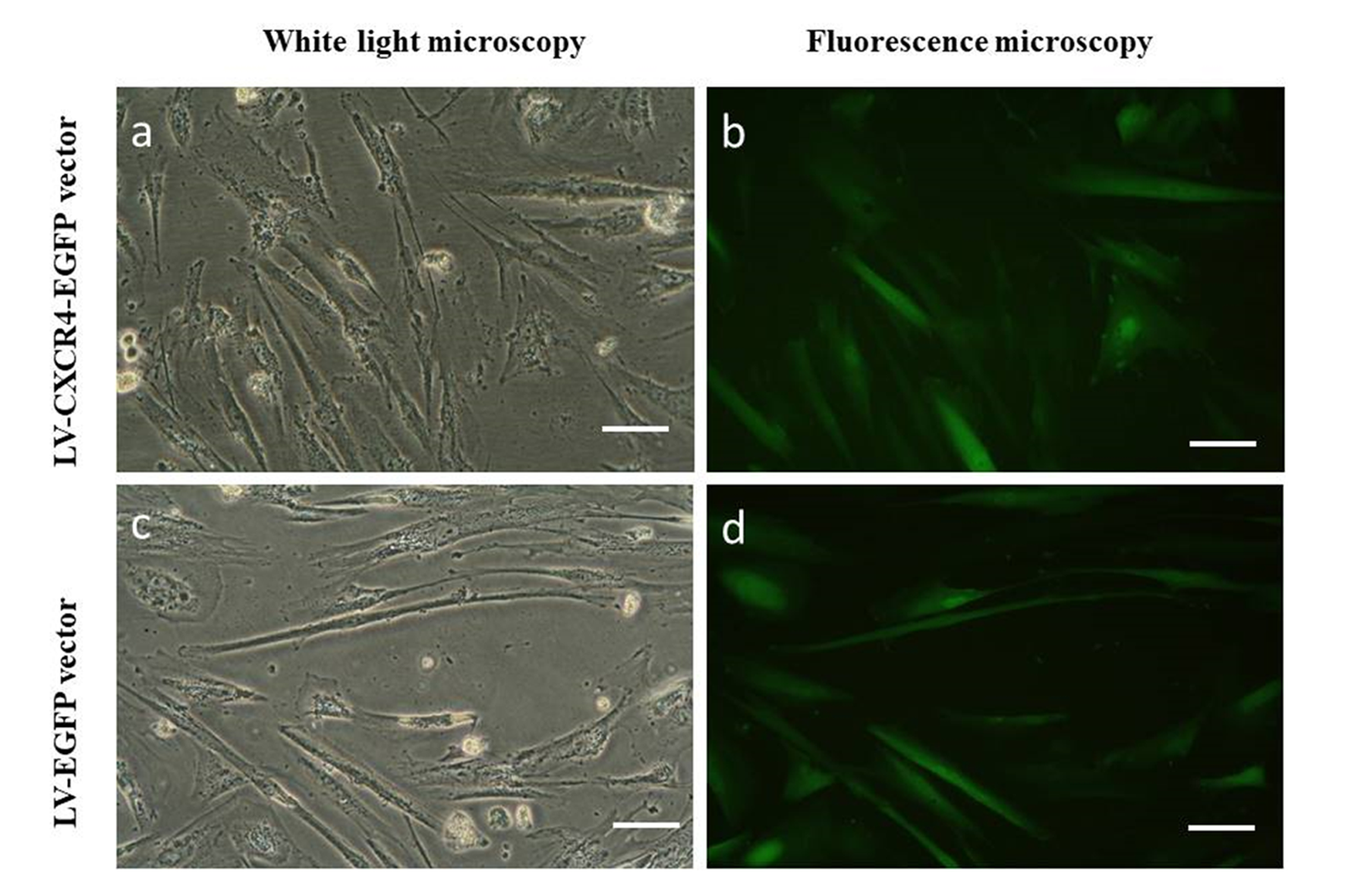

Supplement: Supplementary Materials — Supplementary Figure 1: characterization of HUMSCs. (a) Morphology of HUMSCs. HUMSCs were around the Wharton's jelly fragments at 21 days of culture at 40x (a) magnification (bar = 100 μm). The passage 3 HUMSCs appeared spindle-shaped and reached confluence circinately after 3 days of culture at 40x (b) (bar = 200 μm), and 100x (c) magnification (bar = 100 μm). (b) Immunophenotype of HUMSCs by flow cytometric analysis. The HUMSCs were positive for CD29, CD44, and CD90 and were negative for CD31, CD34, CD45, and HLA-DR. Supplementary Figure 2: transduction of CXCR4 into HUMSCs using a lentiviral vector. The passage 3 cultured HUMSCs after transfection with lentivirus-CXCR4-EGFP vectors (a) or lentivirus-EGFP vectors (c) under white light microscopy at 200x magnification (bar = 50 μm). The expression of EGFP (green) in HUMSCs detected at 96 h after transduction under fluorescence microscopy at 200x magnification; ((b) lentivirus-CXCR4-EGFP vectors; (d) lentivirus- EGFP vectors; bar = 50 μm). Supplementary Figure 3: MTT assay for HUMSCs of CXCR4-overexpressing, control, and normal (P > 0.05, n = 6). [file 2457082.f1.zip › Figure/S Fig2.tif]

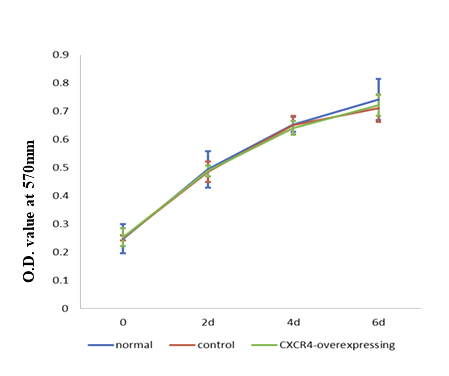

Supplement: Supplementary Materials — Supplementary Figure 1: characterization of HUMSCs. (a) Morphology of HUMSCs. HUMSCs were around the Wharton's jelly fragments at 21 days of culture at 40x (a) magnification (bar = 100 μm). The passage 3 HUMSCs appeared spindle-shaped and reached confluence circinately after 3 days of culture at 40x (b) (bar = 200 μm), and 100x (c) magnification (bar = 100 μm). (b) Immunophenotype of HUMSCs by flow cytometric analysis. The HUMSCs were positive for CD29, CD44, and CD90 and were negative for CD31, CD34, CD45, and HLA-DR. Supplementary Figure 2: transduction of CXCR4 into HUMSCs using a lentiviral vector. The passage 3 cultured HUMSCs after transfection with lentivirus-CXCR4-EGFP vectors (a) or lentivirus-EGFP vectors (c) under white light microscopy at 200x magnification (bar = 50 μm). The expression of EGFP (green) in HUMSCs detected at 96 h after transduction under fluorescence microscopy at 200x magnification; ((b) lentivirus-CXCR4-EGFP vectors; (d) lentivirus- EGFP vectors; bar = 50 μm). Supplementary Figure 3: MTT assay for HUMSCs of CXCR4-overexpressing, control, and normal (P > 0.05, n = 6). [file 2457082.f1.zip › Figure/S Fig3.tif]
